# Regulation of Cell Signaling and Function by Endothelial Caveolins: Implications in Disease

**DOI:** 10.4172/2161-1025.S8-001

**Published:** 2012-01-04

**Authors:** Grzegorz Sowa

**Affiliations:** Department of Medical Pharmacology and Physiology, University of Missouri, Columbia, MO, 65212, USA

**Keywords:** Endothelial cell, Caveolae, Caveolin-1, Caveolin-2, Disease

## Abstract

Caveolae are cholesterol- and glycosphingolipid-rich omega-shaped invaginations of the plasma membrane that are very abundant in vascular endothelial cells and present in most cell types. Caveolins are the major coat protein components of caveolae. Multiple studies using knockout mouse, small interfering RNA, and cell-permeable peptide delivery approaches have significantly enhanced our understanding of the role of endothelial caveolae and caveolin-1 in physiology and disease. Several postnatal pulmonary and cardiovascular pathologies have been reported in caveolin-1 knockout mice, many of which have been recently rescued by selective re-expression of caveolin-1 in endothelium of these mice. A large body of experimental evidence mostly using caveolin-1 knockout mice suggests that, depending on the disease model, endothelial caveolin-1 may play either a protective or a detrimental role. For instance, physiological or higher expression levels of caveolin-1 in endothelium might be beneficial in such diseases as pulmonary hypertension, cardiac hypertrophy, or ischemic injury. On the other hand, endothelial caveolin-1 might contribute to acute lung injury and inflammation, atherosclerosis or pathological angiogenesis associated with inflammatory bowel disease. Moreover, depending on the specific model, endothelial caveolin-1 may either promote or suppress tumor-induced angiogenesis. In addition to overwhelming evidence for the role of endothelial caveolin-1, more recent studies also suggest that endothelial caveolin-2 could possibly play a role in pulmonary disease. The purpose of this review is to focus on how caveolin-1 expressed in endothelial cells regulates endothelial cell signaling and function. The review places particular emphasis on relevance to disease, including but not limited to Pulmonary and cardiovascular disorders as well as cancer. In addition to caveolin-1, possible importance of the less-studied endothelial caveolin-2 in pulmonary diseases will be also discussed.

## Introduction

Caveolae were identified as 50–100 nm omega-shaped, non-coated invaginations of the plasma membrane [1–3]. These organelles are found in most mammalian cell types and tissues, and are particularly abundant in endothelial cells (ECs), adipocytes, and type I pneumocytes [4–6]. The functions originally described for caveolae included cholesterol transport [7,8], endocytosis [9], and potocytosis [10]. However, later studies have revealed that this morphologically distinct subset of lipid rafts plays a pivotal role in regulating cell signaling. Membrane rafts and caveolae concentrate certain membrane proteins and other components involved in transport and signal transduction [11–14].

**Figure 1:**
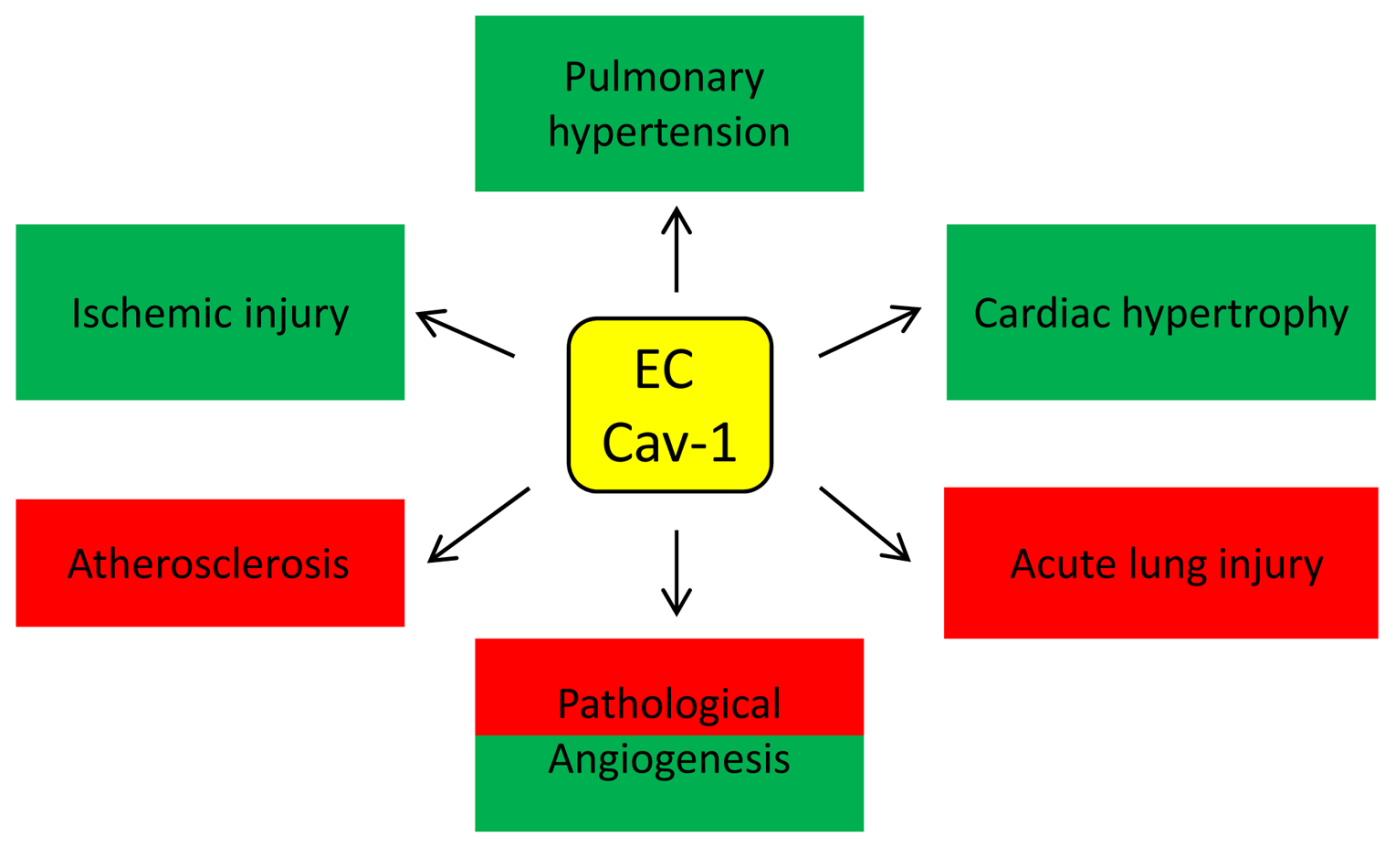
The role of endothelial cell (EC) caveolin-1 (Cav-1) in disease Green indicates pathological processes promoted by a loss of EC Cav-1 in rodent models of disease or in patients with pulmonary hypertension. Thus, approaches restoring or mimicking Cav-1 expression in ECs from patients with pulmonary hypertension might have therapeutic potential. Red represents pathological processes suppressed by a loss of endothelial Cav-1 in mouse models of disease. Thus, approaches suppressing Cav-1 expression or antagonizing Cav-1 function in ECs could potentially alleviate pathological processes such as atherosclerosis, or acute lung injury, as well as pathological angiogenesis associated with tumor growth and inflammatory bowel disease.

A significant advance in understanding the roles of caveolae was revealed by identification of the coat proteins of caveolae: caveolins, VIP21/caveolin-1 (Cav-1), caveolin-2 (Cav-2), and caveolin-3 (Cav-3) [15–19]. Cav-1 and Cav-2 are expressed in most cell types including all cell types of the cardiovascular system, while Cav-3 is expressed primarily in vascular smooth muscle, cardiac, and skeletal muscle. Cav-1 expression is essential for the formation of caveolae, whereas the role of Cav-2 can vary depending on cell and tissue type [20–24].This review will first highlight the mechanistic aspects of Cav-1-mediated regulation of EC Signaling and function. Next, the implications of loss or upregulation of Cav-1 in ECs in various pathological conditions such as pulmonary hypertension, cardiac hypertrophy, acute lung injury, atherosclerosis, ischemia, or pathological angiogenesis associated with cancer and inflammation will be discussed (Figure 1). Possible importance of the understudied endothelial Cav-2 in diseases will also be debated.

## Role of Cav-1 in EC signaling and function

All blood vessels are lined by a monolayer of ECs called the endothelium that helps supply nutrients and oxygen to underlying tissues and organs. In ECs, Cav-1 and Cav-1 are primarily found in plasma membrane caveolae. Caveolae are most numerous in the microvascular endothelia of the lung and are relatively infrequent in the highly restrictive microvascular endothelia of the brain, retina, and testes. Interestingly, caveolae are mostly absent in passively leaky blood vessels with sinusoidal endothelia such as the liver [25]. It is important to note that caveolae contain all of the components required for vesicle formation, fission, docking, and fusion with target membranes [26]. Extensive proteomic studies revealed many proteins specifically enriched in EC caveolae [27]. A large number of signaling molecules that regulate vascular ECs localize to lipid rafts/caveolae. These include, among others, receptors e.g., receptor tyrosine kinase (RTK), G-protein-coupled receptors (GPCRs), transforming growth factor-beta (TGF-β) type I and II receptors, certain steroid receptors, low molecular weight and heterotrimeric G-proteins, and “downstream” enzymes and components including endothelial nitric oxide synthase (eNOS) [13,14].

## Endothelial permeability

Integrity of endothelial barrier function is very important and any disruption of this barrier often leads to excessive accumulation of fluid in the interstitium. This disruption is associated with pathological processes such as acute lung injury, inflammation, atherosclerosis, or pathological angiogenesis associated with cancer and inflammation, which will be discussed later. Numerous studies have shown that caveolae, and specifically Cav-1, are involved in the transport of macromolecules via endothelium. The transcytosis of macromolecules was the first role suggested for caveolae [28]. Caveolae have been proposed to facilitate the transport of molecules such as albumin [29], iron-transferrin [30], insulin [31], low-density lipoproteins (LDL) [29], and chemokines [32]. The transcytosis pathway could be essential for the specific and targeted delivery of molecules to certain tissues and organs. For example, the transcytosis pathway is believed to be critical for the capillary ECs forming the blood brain barrier. The transport of albumin has been the most extensively studied event of transcytosis [33]. The transport of albumin is important since this protein can bind and carry various small molecules such as fatty acids and steroid hormones. The transcytosis of albumin was proposed to be mediated by gp60 receptor localizing to caveolae [34] and later caveolae were shown to mediate the endothelial transcytosis of albumin [35,36]. In mice injected with gold-labeled albumin, Cav-1 KO ECs were unable to transcytose albumin, in contrast to WT [36]. Remarkably, contrary to what could be anticipated based on the positive role of caveolae in albumin transcytosis, Cav-1 KO mice had increased permeability to albumin due to opening of the paracellular junctions in endothelia of small veins and capillaries [37], suggesting a possible interaction between transcellular and paracellular pathways in controlling tissue fluid equilibrium. Moreover, similar results were reported in mice treated with Cav-1 siRNA [38]. Although the vascular aberrations reported in Cav-1 KO mice could be adaptive changes in response to loss of Cav-1, recent data suggest the existence of signaling pathways connecting paracellular permeability and transcytosis. Specifically, the increase in permeability to albumin is related to decreased plasma albumin concentration, atypical morphology of tight junctions, and detachment of ECs from the basement membrane, and edema in lungs of Cav-1 KO mice [37]. Treatment of Cav-1 KO mice with eNOS inhibitor abolished the augmenting effect of Cav-1 loss on paracellular permeability [37], implying that Cav-1 might regulate paracellular permeability and adherens junction integrity through eNOS activity. The opening of adherens junctions which was observed in Cav-1 KO endothelium suggests that Cav-1 is essential for adherens junction assembly or maintenance. Recently, a probable mechanistic explanation regarding this phenomenon was provided by Siddiqui et al. [39], who, using Cav-1 KO ECs, observed that loss of Cav-1 lead to hyperactivation of eNOS further leading to generation of NO and peroxynitrite. Subsequently they determined that the GTPase-activating protein (GAP) p190RhoGAP-A was selectively nitrated at Tyr 1105, leading to impaired GAP activity and RhoA activation. Inhibition of eNOS or RhoA restored adherens junction integrity and reduced endothelial hyperpermeability in Cav-1 KO mice. In addition, thrombin also induced nitration of p120- catenin-associated p190RhoGAP-A. Taken together, these data suggest that loss of Cav-1 in ECs lead to eNOS-dependent nitration of p190RhoGAP-A, and subsequently to adherens junction disassembly resulting in increased endothelial permeability. Curiously, in contrast to the previously discussed increased basal permeability, studies of Sun et al. [40] revealed that Cav-1 KO mice were resistant to hydrogen peroxide-induced pulmonary vascular albumin hyperpermeability and edema formation. Moreover, hyperpermeability in response to hydrogen peroxide, also observed in Cav-1 KO mouse lung microvessels, was reduced by re- expression of WT, but was not reduced by the phosphorylation-deficient mutant of Cav-1. The increase in Cav-1 phosphorylation induced by hydrogen peroxide was associated with both enhanced albumin transcytosis and reduced transendothelial electric resistance in lung ECs. Hydrogen peroxide-induced phosphorylation of Cav-1 lead to the dissociation of vascular endothelial cadherin/beta-catenin complexes and endothelial barrier disruption. Taken together, these data suggest that Cav-1 phosphorylation-dependent signaling is essential for oxidative stress-induced pulmonary vascular hyperpermeability via transcellular and paracellular pathways. Overall, the results of the numerous studies discussed previously suggest that caveolae and Cav-1 play a critical role in regulating microvascular permeability. Moreover, the regulatory role of caveolae and Cav-1 appear to be complex and context-specific, for example basal versus oxidative stress-induced microvascular permeability.

## eNOS

eNOS plays a central role in regulating cardiovascular and pulmonary functions, and hyper-activation as well as deficiency in eNOS signaling may be associated with endothelial dysfunction which is a hallmark of many pathologies such as pulmonary hypertension, acute lung injury, or atherosclerosis, that will be discussed later. eNOS was one of the earliest non-receptor proteins localized to caveolae [41]. Numerous studies have characterized the interaction between eNOS and Cav-1 in vitro. Specifically, using co-immunoprecipitation and domain-mapping approaches, several groups have shown that eNOS directly binds to the Cav-1 scaffolding domain (CSD; aa 81–101) of Cav-1 [42,43]. Evidence supporting the functional relevance of this interaction in intact cells was shown by delivery of a cell-permeable peptide containing the CSD or by overexpression of Cav-1 in living cells or tissues. In all cases, NO release was reduced, consistent with an inhibitory function of Cav-1 in regulating eNOS activity [44–46]. Moreover, we have demonstrated that Cav-1 present in caveolae but not lipid rafts is capable of inhibiting eNOS under basal but not stimulating conditions [46]. This suggests that not only lipid raft but specific caveolar localization is necessary for optimal tonic inhibition of eNOS by Cav-1. Subsequently, several studies using a Cav-1 KO approach reinforced the negative regulation of eNOS by caveolae and Cav-1. Specifically, the two independent studies reporting original phenotypes in Cav-1 KO mice, revealed that the basal NO release and cGMP production were both significantly higher in Cav-1 KO compared to WT mice [47,48]. These data clearly indicate that loss of Cav-1 and caveolae results in hyperactivation of eNOS and associated NO release. Moreover, in addition to eNOShyperactivation, a lack of steady contractile tone in Cav-1 KO aortas, as well as an increased relaxation in response to acetylcholine was reported. This was accompanied by a lower L-NAME-sensitive steady-state maximal tension in response to phenylephrine. These data clearly indicate that Cav-1 and caveolae are critical for the negative regulation of eNOS activity in vivo. Recent data suggest that antioxidants such as resveratrol can enhance the generation of NO as a consequence of an increased phosphorylation and activity of eNOS in ECs [49]. Notably, in addition to NO that stimulates signaling via activation of PKG, local activation of eNOS can also lead to S-nitrosylation of proteins [50]. In contrast to the stimulating function of free NO, protein nitration was later shown to play a negative role in cell signaling and function. For instance, recent studies of Zhao et al. [51] have shown that eNOS hyperactivation observed in Cav-1 KO mice results in excessive peroxynitrite production and inhibitory nitration of PKG, leading to pulmonary hypertension.

## Intracellular calcium

Abnormal calcium-ion transport in ECs is often associated with endothelial dysfunction. Several proteins participating in calcium-ion transport are targeted to caveolae and are regulated by Cav-1. Moreover, caveolar localization and/or interaction with Cav-1 may be required for proper functioning of certain calcium-ion channels in various cell types including ECs [52]. Pertinent to ECs, studies of Isshiki et al. [53] demonstrated that calcium waves originate in endothelial caveolae. In addition, Cav-1 was shown to regulate store-operated calcium-ion influx via binding via CSD to transient receptor potential channel-1 (TRPC1) in ECs [54]. Studies by Murata et al. [55] showed that loss of Cav-1 expression in ECs abrogated calcium-ion entry due to calcium-ion store depletion as a consequence of impaired protein-protein interactions between TRPC1 and 4, and their targeting to plasma membrane lipid rafts. They also showed that re-expression of Cav-1 in Cav-1 KO ECs restored normal calcium-ion entry, TRPC1 and 4 interactions and their targeting to plasma membrane lipid rafts [55]. Taken together, these data suggest that Cav-1 plays the crucial function in maintaining proper calcium-ion entry in ECs.

## Redox signaling and function

Abnormalities inredox signaling play important role in EC dysfunction and associated diseases, such as inflammation or acute lung injury. Experimental evidence suggests that the NADPH Oxidase (NOX) complex is preassembled and functional in caveolae and its enzymatic activity is further enhanced by recruitment of additional components to caveolae [56]. Numerous stimuliorganize NOX components in ceramide-enriched lipid rafts [57,58]. Moreover, assembly of such complexes is often initiated by pro-apoptotic signals including Fas ligand or TNF-α [59]. Apart from pro-apoptotic signals, lipolysis of triglyceride-rich lipoproteins can lead to aggregation of lipid rafts and enhancement of ROS production in ECs [60]. Cav-1 was shown to function as a sensor of shear stress in ECs and to regulate ROS-mediated signaling via NOX [61]. Proximal NO and superoxide production by eNOS and NOX, respectively, might possibly contribute to protein nitration on tyrosine residues. Disruption of lipid raft/ caveolae microdomains with cholesterol-sequestering drugs dissociates these enzymes from lipid Raft /caveolar domains, resulting in reduced protein nitration in bovine aortic ECs [56].

The Heme Oxygenase (HO) family, consists of three membrane bound members: inducible HO-1, constitutive HO-2, and HO-3 which is catalytically inactive [62]. Among the products generated by HO, carbon monoxide (CO) has been implicated in cell signaling [63]. It was also shown that CO can be regulated by NO and potentially mediate vascular function [64, 65]. In addition, HO-1 was shown to interact with Cav-1 and Cav-2, localize to caveolae and to be negatively regulated by Cav-1 [66]. Importantly, genetic evidence suggests that loss of Cav-1 in mice protects these animals from hyperoxic damage in the lung due to an increased expression and activity of HO-1 [67].

## Mechanotransduction

The endothelium is always exposed to mechanical forces regulating its function [68]. It is well recognized that laminar and disturbed flows regulate endothelial function differently. For instance, disturbed flow is associated with atherosclerosis or tumor blood vessels. Initially, studies by Park et al. [69] revealed that exposure of ECs to shear stress increased number of caveolae. Moreover, Rizzo et al. [70] showed that shear stress can also stimulate NO production in ECs by promoting the dissociation of eNOS from Cav-1. In another study from the same group Rizzo et al [71] demonstrated that exposure of ECs to shear stress could also lead to tyrosine phosphorylation of caveolar proteins. Studies of Sun et al. [72] have also shown the translocation of numerous signaling molecules to caveolae, subsequently leading to activation of the Ras-p42/44/MAPK pathway. Studies involving Cav-1 KO mice, and ECs isolated from these mice, further reinforced the functional significance for Cav-1 in short- and long-term mechano transduction in the vasculature [73]. Most recently, Yang et al. [74] showed that p190RhoGAP links integrins and Cav-1/caveolae to RhoA in a mechano transduction cascade that participates in endothelial adaptation to flow. Taken together, these data suggest that EC caveolae in general, and Cav-1 in particular, plays essential roles in controlling normal endothelial response to shear stress and vascular flow in vitro and in vivo.

## Endothelial Cav-1 and Disease

Numerous pathologies have been reported in Cav-1 KO mice, many of which are likely a consequence of a selective loss of Cav-1 in endothelium. Remarkably, specific re-expression of Cav-1 in endothelium of Cav-1 KO mice reversed most of pulmonary and cardiac pathologies, suggesting that loss of Cav-1 in endothelium might be rimarily responsible for both pulmonary and cardiac defects. In addition, endothelial Cav-1 was shown to play an important role in other pathologies such as atherosclerosis, tissue ischemia, lung injury, or pathological angiogenesis and inflammation (Figure 1).

## Endothelial Cav-1 and pulmonary hypertension

Endothelial dysfunction, cell hyper-proliferation and impaired apoptosis are key features of pulmonary hypertension. Numerous cardiopulmonary, autoimmune and inflammatory diseases, portal hypertension, or exposure to appetite suppressants are known to lead to pulmonary hypertension [75]. Regardless of its etiology, pulmonary hypertension is associated with endothelial dysfunction, medial hypertrophy, neointima formation, occlusion of small arteries leading to elevated pressure, and right ventricular hypertrophy with subsequent right heart failure and premature death. Several independent studies have demonstrated pulmonary arterial hypertension in Cav1 KO mice [76–78]. Moreover, marked reduction of endothelial Cav-1 has been reported in a number of clinical and experimental forms of pulmonary hypertension [79, 80, 81]. For instance, Cav1 reduction can be seen in rat models of pulmonary hypertension including monocrotaline-induced pulmonary hypertension [79, 80] and U5416/hypoxia-induced pulmonary hypertension [82]. Specifically, Cav1 (predominantly the EC-restricted isoform Cav1α in the lung) expression was decreased in rat lungs starting 48 h after monocrotaline challenge [80]. Intriguingly, the reduction of Cav-1 expression was mainly seen in the intimal layer of the pulmonary arteries but not the pulmonary veins. Moreover, reduced Cav1 expression was associated with hyperactivation of STAT3 and ERK1/2 signaling in pulmonary ECs upon treatment with monocrotalinepyrrole in vivo and in vitro [80]. Interestingly, in addition to rats with chemically-induced pulmonary hypertension, a similar increase in tyrosine phosphorylated STAT3, and the expression levels of cyclin D1 and D3 were observed in whole lung homogenates from Cav-1 KO mice [79]. Importantly, delivery of a cell permeable peptide containing CSD reversed pulmonary hypertension and the accompanying increases in pulmonary phospho-STAT3, cyclin D1, and cyclin D3 expression in rats exposed to monocrotaline [83]. In another rat model of severe pulmonary hypertension, induced by a single subcutaneous injection of the VEGFR inhibitor SU5416 and subsequent 3-week exposure to chronic hypoxia, Cav1 expression was selectively reduced in the arterial lesions [82]. Furthermore, these authors also observed decreased expression of Cav-1 in lungs of patients with severe pulmonary hypertension. Specifically, the reduced expression levels of Cav1 could be seen in plexiform lesions and in some muscularized precapillary arterioles in lung tissues without ignificant change in Cav-1 expression in total lung lysates [82]. In contrast to the previously discussed findings, a study by Patel et al. [84] found decreased expression of Cav-1 in total lung lysates. In addition, they also observed decreased expression of Cav-1 in pulmonary vascular ECs in lung tissues from idiopathic pulmonary aortic hypertension patients. Remarkably, in contrast to ECs, Cav1 expression was elevated in smooth muscle cells in lung tissues from idiopathic pulmonary aortic hypertension patients. These data suggest differential regulation of Cav-1 expression levels between ECs and smooth muscle cells during pulmonary hypertension in humans.

Consistent with results reported earlier, recent studies also demonstrated a marked decrease of Cav-1 expression in total lung lysates from idiopathic pulmonary aortic hypertension patients [51]. Altogether, the results of these studies, strongly suggest that Cav-1, particularly as expressed in pulmonary arterial ECs, is a critical regulator of pulmonary vascular function and that specific reduction of Cav-1 in pulmonary arterial vessels could play an important role in the pathology of pulmonary hypertension in humans.

Since eNOSis an important target of Cav-1, it is not surprising that numerous studies explored the possibility that changes in eNOS activity and function could be responsible for the pulmonary hypertension seen in Cav-1 KO mice. For example, in one study, treating Cav-1 KO mice with the NOS inhibitor l-NAME in the first two months of life, resulted in a complete reversal of pathological pulmonary changes, heart hypertrophy and pulmonary arterial hypertension [77]. Moreover, they also found evidence for enhanced oxidative stress in Cav-1 KO mice that was substantially reduced by chronic l-NAME treatment. Based on this data, the authors suggested that a perturbation of NO signaling, together with enhanced superoxide production originating from NO synthases, could play a pivotal role in the pathogenesis of the pulmonary arterial hypertension seen in Cav-1 KO mice.

In a subsequent study the same group tested the hypothesis that eNOS uncoupling, with generation of free radicals superoxide, peroxinitrite drives the cardiopulmonary changes in Cav-1 KO mice [85]. Specifically, they determined that the Cav-1 KO vessels produced more superoxide, and that the ratio between the essential eNOS cofactor tetrahydrobiopterin (BH4) and its precursor and oxidation product dihydrobiopterin was reduced. This reduction in BH4 would most likely uncouple NOS activity, resulting in increased superoxide production. Finally, dietary supplementation with BH4 restored the BH4/dihydrobiopterin ratio and normalized cardiopulmonary phenotype. Altogether, these data strongly supports the idea that loss of Cav-1 uncouples eNOS resulting in free radical production, tissue damage and remodeling.

Consistent with Wunderlich’s observations, in another study, using a double knockout of Cav-1 and eNOS (Cav-1/eNOS KO), Zhao et al. [51] have shown that Cav-1/eNOS KO mice did not develop pulmonary hypertension and the ratio of right ventricle weight/left ventricle plus septum weight was normalized in Cav-1/eNOS KO mice. The defects in pulmonary vasculatures and alveolar capillary structures as well as vessel wall thickness were all corrected in Cav-1/eNOS KO mice. In addition to data with mouse models, they also performed studies on samples from idiopathic pulmonary arterial hypertension patients. Interestingly, they observed increased eNOS activity and PKG nitration concomitant with decreased Cav-1 expression in lung tissues from idiopathic pulmonary arterial hypertension patients compared with normal lungs in the absence of marked changes of eNOS and PKG expression [51]. These findings are in agreement with previous studies showing that decreased Cav-1 expression in lungs with idiopathic pulmonary arterial hypertension is mainly in the plexiform lesions [82] and selectively in ECs [84]. Because eNOS is robustly expressed in the plexiform lesions of idiopathic pulmonary arterial hypertension lungs [86], these data support the idea that eNOS hyperactivation due to loss of Cav-1 is important in developing pulmonary hypertension in mice and humans. Moreover, PKG nitration and subsequent impairment of its kinase activity is a critical downstream target through which hyperactive eNOS induces pulmonary hypertension in mouse models and in humans.

Altogether, the data discussed in this chapter also strongly suggest that loss of Cav-1 in ECs is primary responsible for the pulmonary hypertension observed in Cav-1 KO mice and in humans. This notion is further supported by the fact that pulmonary hypertension was completely reversed in Cav-1 KO mice by a specific re-expression of Cav-1 in endothelium [76]. Thus therapeutic approaches restoring Cav-1 expression in endothelium, using cell-permeable CSD peptide, could potentially be useful in treatment of pulmonary hypertension.

## Is loss of Cav-1 expression in ECs the causative factor for cardiac hypertrophy?

Cardiac hypertrophy is a critical pathology leading to heart failure. Although ventricular cardiomyocytes express primarily Cav-3, surprisingly numerous studies have reported cardiomyopathy in Cav-1 KO mice [76,78,87,88] For example, Cohen et al. [87] have shown that Cav-1 KO mice develop progressive concentric left ventricular hypertrophy, as well as right ventricular dilation. Cav-l expression was restricted to the supporting cells of the heart, such as fibroblasts and ECs. Excessive activation of the Ras-p42/p44-MAP kinase cascade could be seen in Cav-l KO cardiac fibroblasts and thus the authors concluded that these changes in fibroblasts are important upstream factors promoting hypertrophy and fibrosis in the adjacent myocytes. Moreover, Cav-1 KO cardiac fibroblasts exhibited p42/p44 MAP kinase hyperactivation as compared to WT fibroblasts. This data suggests that the hypertrophy observed in Cav-l KO hearts is a result of a paracrine regulation of myocytes fibrosis by hyperproliferating fibroblasts [87]. However, later studies by Murata et al. [76] revealed that the mechanism leading to cardiac hypertrophy in Cav-1 KO mice are even more indirect and appear to originate in ECs rather than fibroblasts. Specifically, they showed that selective re-expression of Cav-1 in endothelium completely reversed cardiac hypertrophy and associated fibrosis [76]. Perhaps, additional studies using an independent EC-specific promoter such as VE-cadherin are needed to further confirm the importance of endothelial Cav-1 in cardiac pathophysiology.

## The role of endothelial Cav-1 in acute lung injury and associated inflammation

Primary acute lung injury is a direct injury to the lung which may be caused by pneumonia, ventilation-associated injury, hyperoxic injury, trauma, and contusion [89–91]. Secondary acute lung injury can be caused indirectly by conditions such as severe sepsis, pancreatitis, or transfusion-related acute lung injury [89–91]. Acute inflammation has been associated with the pathological stages of acute lung injury and acute respiratory distress syndrome, along with augmented vascular permeability, fibroproliferation, epithelial cell apoptosis, and varying degrees of interstitial fibrosis [89–91]. Results of several studies suggest that caveolae and Cav-1 expressed in ECs play a critical role during acute inflammation, primarily through regulating the transport of macromolecules such as albumin from the blood-space to the tissue-space [37,40,92,93]. Albumin is not endocytosed by Cav-1 KO lung ECs and remains in the blood vessel lumen [40]. Caveolae are particularly abundant in ECs and transmembrane water channel protein aquaporin-1 is expressed in caveolae of lung ECs [94], suggesting that caveolae play a critical role in endothelia-mediated transcellular transport of water [94]. Previous studies have suggested that caveolae and lipid rafts contribute to non-cardiogenic pulmonary edema during acute lung injury [92,93]. Later studies with a specific targeting of Cav-1, using a cell-permeable CSD peptide have shown that CSD regulates calcium store release-induced calcium influx in ECs, suggesting a potential role in endothelial permeability [95]. Evidence has also been gathered suggesting that Cav-1 may play a dual role in regulating microvascular permeability: i.e As a caveolae-associated structural protein, Cav-1 may control caveolar transcytosis; ii. As a tonic inhibitor of eNOS activity, Cav-1 may negatively regulate paracellular permeability [40]. As a result of this dual regulation, although the Cav-1 KO mice have lung vascular and fluid balance abnormalities, these mice are resistant to acute lung injury relative to WT mice. Moreover, Cav-1 KO mice have improved survival following LPS challenge [96,97]. Similar results were reported using hyperoxia [67]. Remarkably, Cav-1 KO mice have basal pulmonary edema, with elevated extravascular lung water. It has been postulated that this opposing tissue pressure may limit further transport and accumulation of pulmonary edema fluid because of vascular damage during lung injury [98]. In addition, loss of Cav-1 leads to hyperactivation of eNOS and subsequent dampening of Tolllike receptor 4 (TLR4) signaling, resulting in decreased innate immune response to LPS that protects from LPS-induced inflammation and injury [97]. These data are consistent with the previous studies by Garrean et al. [96], showing that Cav-1 KO mice have an impaired inflammatory response to LPS via NF-κB-mediated pathways. Taken together, the literature discussed in this chapter suggests that Cav-1 expressed in lung microvascular ECs may promote acute lung injury in mice. However, EC-specific re-expression of Cav-1 in Cav-1 KO mice or EC-specific Cav-1 KO approaches will be required to unequivocally determine if loss of endothelial Cav-1 is entirely responsible for the protection from lung injury observed in global Cav-1 KO mice.

## Endothelial Cav-1 and atherosclerosis

Atherosclerosis is the result of inflammatory and fibro-proliferative responses which reflect a complex crosstalk among the vascular wall, circulating cells and cardiovascular risk factors. Many studies involving animals and humans have provided the evidence showing that EC dysfunction plays a major role in initiation of the atherosclerotic process [99, 100]. Substantial reduction in the production, bioavailability, and actions of eNOS is one of the major outcomes of EC dysfunction [99,101]. Cardiovascular risk factors (high cholesterol, smoking, diabetes, and others) disturb the normal equilibrium of the vascular wall, thereby stimulating the expression of adhesion molecules such as E-selectin, P-selectin, ICAM-1 and VCAM-1 [102]. As a consequence, circulating leukocytes are attracted to endothelium, contributing to an “inflammatory state.” These events are driven by the deposition of low density lipoprotein (LDL) and their modification in the subendothelial space [103]. The entrapment of LDL particles in the sub-endothelial space of arteries and their subsequent modification is believed to be one of the key events that ultimately lead to the development of an atheroma [104,105].

Alarge body of evidence suggests that endothelial caveolae and Cav-1 have the potential to affect the process of atherosclerosis. Cav-1 is a cholesterol-binding protein that can transport cholesterol from the endoplasmic reticulum to the plasma membrane. Moreover, the major receptors for high-density lipoprotein, SR-B1, and a scavenger receptor for modified forms of LDL, CD36, can localize to caveolae microdomains [106]. In addition, oxidized LDL can extract caveolar cholesterol, mislocalize eNOS, and impair NO release [107]. Conversely, blockade of HMG CoA reductase with statin-based drugs reduced Cav-1 expression levels and led to eNOS activation [108]. In apolipoprotein (ApoE) KO mice, treatment with statin decreased Cav-1 expression and increased eNOS activity in vivo. Results of numerous studies suggest that Cav-1has a pro-atherogenic function. For example, Cav-1 is upregulated in ECs upon LDL exposure [109]; down-regulation of Cav-1 is associated with reduced uptake of oxidized DL by ECs [110]. Cav-1 translocation to the plasma membrane is enhanced in ECs incubated with LDL. It was shown that oxidized LDL can modify the localization of both Cav-1 and eNOS, resulting in impaired eNOS activation by acetylcholine, most likely due to disruption of the signaling complex containing eNOS and other molecules required for eNOS activation [109]. In another study, it was demonstrated that CD36, a class B scavenger receptor which is associated with caveolae, could possibly be responsible for this effect [110]. This result is important because impairment of endothelium-derived relaxation is observed in both hypercholesterolemic patients and animal models [111, 112]. Consistent with these findings, it was also shown that exposure of ECs to serum from hypercholesterolemic patients increases the interaction between eNOS and Cav-1 [113].

Although the findings of the above mentioned studies strongly support the importance of caveolae and Cav-1, the direct confirmation has been obtained in Cav-1 KO mice. Specifically, using Cav-1 KO mice bred to ApoE KO mice, Frank et al. [114] showed that the loss of Cav-1 reduces fatty streak lesion formation by c.a. 70%, compared with ApoE KO mice. This reduction was associated with lower CD36 expression in the aorta. Moreover, plasma LDL levels in Cav-1 KO mice were increased, suggesting a problem with either uptake and/or transfers of LDL to peripheral tissues, which is consistent with a role for caveolae in the LDL transcytosis process [114]. Because, in addition to ECs, macrophages are also involved in LDL uptake, it was important to determine a specific contribution of ECs to this process. To address this, an EC-specific reexpression of Cav-1 in Cav-1 KO mice was used [115]. These studies revealed that although global loss of Cav-1 in an ApoE KO background inhibited atherosclerotic lesion expansion, EC-specific re-expression of Cav-1 restored this process. Mechanistically, loss of Cav-1 reduced LDL infiltration into the arterial wall, promoted NO production, and reduced the expression of leukocyte adhesion molecules such as VCAM-1, ICAM-1 and E-selectin. The latter effects of global loss of Cav-1 were completely reversed by re-expression of Cav-1 in endothelium [115]. Subsequent studies from the same laboratory also determined that endothelial-specific overexpression of Cav-1 enhanced the progression of atherosclerosis in mice which was associated with reduced EC proliferation, migration, and NO production in vitro and increased expression of VCAM-1 in vivo [116]. Taken together, these data strongly suggest that Cav-1 expressed in ECs plays a pro-atherogenic role in mouse models of atherosclerosis. Interestingly, the clinical evidence obtained to date suggests that Cav-1 could play an anti-atherogenic role in humans. Specifically, studies of Rodriguez-Feo et al. [117] revealed that the expression levels of Cav-1 were significantly lower in carotid plaques than non-atherosclerotic vascular specimens harvested from a large group (378) of subjects that underwent carotid endarterectomy. Also, low expression levels of Cav-1 were associated with plaque instability. Taken together, these results suggest that local down-regulation of Cav-1 in atherosclerotic lesions might contribute to plaque formation and/or instability leading to an increased occurrence of adverse clinical outcomes. Furthermore, loss of Cav-1 could be considered as a biomarker of vulnerable plaque with prognostic value [117]. Overall, the data using cultured ECs, mouse models of atherosclerosis provide a compelling mechanistic evidence for pro-atherogenic role of Cav-1. However, the mechanistic aspects of the involvement of Cav-1 in human atherosclerosis remain to be elucidated.

## Endothelial Cav-1 and pathological angiogenesis associated with cancer and inflammation

Angiogenesis is a process of new blood vessel formation that takes place in three clearly distinct phases: initiation, proliferation of ECs, and morphogenesis. ECs play the central role in the process of angiogenesis. In contrast to the normal vasculature, the new blood vessels that develop in response to tumors and other pathological stimuli such as chronic inflammation are non-uniformly distributed, branch irregularly, do not conform to a clear hierarchical pattern, and are hyperpermeable to plasma and plasma proteins. Several important signaling proteins involved in angiogenesis have been localized to caveolae such as the VEGF receptor (VEGFR), the urokinase receptor (uPAR), eNOS, TGF-β receptors.

Experimental evidence has accumulated suggesting that, depending on specific context, Cav-1 may play either a positive or negative role in pathological angiogenesis. Original studies using WT and Cav-1 KO mice implanted with basic fibroblast growth factor-loaded Matrigel plugs revealed reduced angiogenesis in Cav-1 KO mice [118], implying that Cav-1 is required for optimal postnatal angiogenesis. In addition, three independent studies that used in vivo models of tumor-induced angiogenesis involving mouse B16 melanoma cells implanted in Cav-1 KO and WT C57BL/6 mice [118,119] and RM-9 prostate cancer cells or human prostate cancer LNCaP cells implanted into nude mice [120], have determined that Cav-1 plays a pro-angiogenic role in tumor-induced angiogenesis. Specifically, in the first study Woodman et al. [118] showed reduced tumor weight, volume, and vessel density in Cav-1 KO mice injected subcutaneously (s.c.) with B16 melanoma cells.

Similar results with B16 melanoma implanted in Cav-1 KO mice were reported later by Chang et al. [119], reinforcing the idea that Cav-1 plays a pro-angiogenic role in B16 melanoma-induced angiogenesis in mice. The pro-angiogenic role of Cav-1 was also reported by Tahir et al. [120], who observed reduced tumor growth and angiogenesis in Cav-1 KO mice using an orthotopic RM-9 mouse prostate cancer model. Altogether, these the above discussed data suggest that Cav-1 expressed in host environment, plays a positive role in tumor-induced ngiogenesis in vivo. Is Cav-1 expressed in ECs primarily responsible for the positive role of Cav-1 in pathological angiogenesis in vivo? Although ECs play a central role in the process of tumor-induced angiogenesis, it is also important to remember that other host cells such as stromal cells could indirectly modulate the process of angiogenesis. Although not done for tumor-induced angiogenesis, the direct role of Cav-1 expressed in ECs was recently addressed by Chidlow et al. [121] in another murine model of pathological angiogenesis associated with inflammatory bowel disease. Specifically, angiogenesis was markedly reduced in global Cav-1 KO mice relative to WT mice with pharmacologically-induced colitis. Remarkably, specific re-expression of Cav-1 in the endothelium of Cav-1 KO mice resulted in increased angiogenesis which was comparable to the result in WT mice. These data clearly suggest that Cav-1 expressed in ECs plays a positive role in pathological angiogenesis associated with experimental colitis. Thus, Cav-1 expressed in ECs could be considered as a potential therapeutic target for inflammatory bowel disease.

In addition to the previously discussed role of Cav-1 in pathological angiogenesis in vivo, numerous studies involving isolated primary ECs also suggest that Cav-1 plays a positive role in angiogenesis in vitro. Specifically, at least two studies including Cav-1 KO aortic EC support the pro-angiogenic role for Cav-1 in vitro. For example, studies of Sonveaux et al. [122] showed that both VEGF-induced signaling and angiogenesis were suppressed in Cav-1 KO aortic ECs compared to WT counterparts. Consistent with the aforementioned studies, Tahir et al. [120] showed that treatment with recombinant Cav-1 restored angiogenic functions in Cav-1 KO aortic ECs. A number of studies involving downregulation of Cav-1 with antisense or siRNA, often combined with Cav-1 overexpression, also support pro-angiogenic function of Cav-1 in cultured human ECs. Specifically, antisense oligos-mediated knockdown of Cav-1 resulted in suppression of capillary tube formation in fibrin gel-based angiogenesis assay [123]. Liu et al. [124] showed that overexpression of Cav-1 or treatment with cell-permeable CSD peptide increased capillary-like tube formation in matrigel, while down-regulation of Cav-1 with antisense decreased capillary-like tube formation in matrigel. Studies of Galvez et al. [125] revealed that caveolae-disrupting agents cyclodextrin or filipin, or siRNA knockdown of Cav-1 decreased MT1-MMP function, cell migration through polycarbonate filters, invasion into collagen gel, and capillary tube formation in matrigel. Consistent with the latter studies, Beardsley et al. [126] showed that siRNA knockdown of Cav-1 also inhibited directional EC migration in response to VEGF, suggesting that Cav-1 is essential for VEGF-induced migration. Taken together, compelling experimental evidence has accumulated suggesting that endothelial Cav-1 may promote pathological angiogenesis associated with cancer and inflammation.

Some of the clinical studies also seem to support the positive role of Cav-1 in tumor-induced angiogenesis (in particular studies showing a positive correlation between Cav-1 expressions), tumor microvascular density, and often shorter survival time. For instance, Joo et al. [127] showed that there was a good correlation between microvascular density and Cav-1-specific immunostaining. Their studies were performed on clear cell renal cell carcinoma tissue sections from 67 patients undergoing radical nephrectomy, using double immunohistochemical staining with specific antibodies against Cav-1 and the EC-specific marker CD34. The higher intensity of Cav-1/CD34 co-immunostaining significantly correlated with the degree of metastasis and significantly worse survival of patients [127]. These data suggest that Cav-1 expressed in tumor ECs might play a pro-angiogenic role in the progression of clear cell renal carcinoma, resulting in a poor clinical prognosis for patients.

Using double immunofluorescent labeling with antibodies to CD34 and Cav-1, Yang et al. [128] determined that the microvascular density values were also significantly higher in Cav-1-positive than in Cav-1-negative prostate cancer tumors. Importantly, they also observed an increased Cav-1 positivity in tumor-associated ECs, primarily restricted to regions with Cav-1-positive tumor cells, corresponding to the higher percentage of Cav-1-positive microvessels within these regions, as opposed to Cav-1-negative tumors. This data suggest that Cav-1 released by prostate cancer cells could play a pro-angiogenic role during prostate cancer progression in humans [128]. Using dual-label immunofluorescence staining with Cav-1 and CD34 antibodies in hepatocellular carcinoma sections Zhang et al. [129] showed a positive correlation between Cav-1 expression and microvascular density, implying that Cav-1 plays a positive role in regulating hepatic cell carcinoma tumor-induced angiogenesis in humans. Taken together, the results of these clinical studies support pro-angiogenic role of Cav-1 expressed either in tumor endothelial cells or tumor cells themselves.

There is also experimental evidence suggesting that Cav-1 could play an anti-angiogenic role. An anti-angiogenic function for Cav-1 has been shown using Cav-1 KO, Cav-1 overexpression or delivery of cell permeable CSD peptide. In the first study, Brouet et al. [130] showed that in vivo transfection of Cav-1 delayed Lewis lung carcinoma (LLC) tumor growth in mice. Consistent with Brouet’s study, Lin et al. [131] showed increased tumor growth, angiogenesis, and permeability in Cav-1 KO mice with subcutaneously implanted LLC tumors. Moreover, treatment with cell-permeable CSD peptide prevented increased tumor microvessel permeability and tumor growth in Cav-1 KO mice. Taken together, these data suggest that Cav-1 may play anti-angiogenic role in a murine LLC model of tumor-induced angiogenesis. Consistent with the Lin’s study using LLC, increased tumor growth, angiogenesis and tumor vessel permeability were observed in Cav-1 KO mice subcutaneously implanted with B16 melanoma cells [132]. At this point, it is not clear as to why results obtained in this study are opposite of previously discussed studies also involving a B16 melanoma model of tumor-induced angiogenesis [118,119].

To date, limited clinical evidence is available to support a potential negative role for tumor EC-expressed Cav-1 in tumor-induced angiogenesis in humans. Specifically, Shi et al. [133] using immunohistochemical labeling with antibodies against Cav-1, VEGF, and CD34 in patients with mucoepidermoid carcinoma, showed an inverse correlation between increased microvascular density and the expression levels of Cav-1 in tumor microvasculature. These data suggest that decreased expression of Cav-1 and increased microvascular density might translate into a poor prognosis for patients with mucoepidermoid carcinoma.

It may be important to reconcile studies showing a pro-angiogenic role of Cav-1 with those studies indicating an anti-angiogenic role. One of the features of angiogenesis is the balance between pro- and anti-angiogenic factors that may depend on a specific tumor model, phase of tumor growth and angiogenesis, genetic backgrounds, age, or gender of animals used for experiments. Moreover, variations among specific angiogenesis assays in vitro, EC source, or pro-angiogenic stimuli may lead to different end result. Numerous studies showed that Cav-1 via its scaffolding domainis capable of interacting with and inhibiting function of several signaling molecules such as eNOS, PI3K, Src, PKC, or Erk that play a role in angiogenesis (see review by [14]. It is likely that when no, or low levels of pro-angiogenic stimuli are present, Cav-1 has an anti-angiogenic function. However, once a critical level of proangiogenic stimulation is achieved, the functional role of Cav-1 could switch from anti- to pro-angiogenic. This notion could be supported by the fact that Cav-1 is essential for maintaining intact and functional caveolar membranes. In addition, caveolar targeting may be essential for optimal functional activity of many receptors and downstream signaling proteins involved in angiogenesis such as VEGFR2 [122, 134], PDGF receptor, Src, eNOS, PI3K, or PKC [135]. While the previous studies may explain differences between n various models or stages of angiogenesis, it is very difficult to explain the opposite outcome of tumor-induced angiogenesis involving the same model such as B16 melanoma. The most likely explanation seems to be different KO-targeting approaches and background in which these mice were generated. EC-specific re-expression of Cav-1 in Cav-1 KO mice, such as that done in Chidlow’s murine model of pathological angiogenesis associated with drug-induced experimental colitis [121] could possibly help to explain these differences.

## Endothelial Cav-1 and Ischemia

Recent studies have directly addressed the functional role of Cav-l expressed in ECs in ischemic injury [122,136]. Initially, Sonveaux et al. [122] used Cav-1 KO mice to examine the role of Cav-1 in a hindlimb ischemia model. Specifically, evaluation of the ischemic tissue perfusion and histochemical analyses revealed that in contrast to WT, Cav-1 KO mice were unable to recover a functional vasculature and lost part of the ligated limbs. ECs isolated from Cav-1 KO aorta displayed an impaired response to VEGF stimulation, NO production and endothelial tube formation. Cav-1 transfection in Cav-1 KO aortic ECs redirected the VEGFR-2 to caveolarmembranes and restored the VEGF-induced ERK and eNOS activation. In another study, Jasmin et al. [136] examined the role of Cav-1 in the pathogenesis of cerebral ischemia. Initially, using immunoblotting and immunofluorescence analyses of rat brains subjected to middle cerebral artery occlusion, they have shown increases in the expression levels of Cav-1 and Cav-2 proteins in ECs. Next, they investigated the effects of cerebral ischemia in Cav-1 and Cav-2 KO mice and have shown marked increase of cerebral volume of infarction and elevated apoptotic index in Cav-1 KO relative to WT and Cav-2 KO mice. Importantly, there was a reduced number of proliferating ECs in Cav-1 KO ischemic brains as compared to WT counterparts. Similarly, the expression levels of eNOS were markedly reduced in Cav-1 KO ischemic brains as compared to WT counterparts. These data suggest that Cav-1 expressed in ECs plays a protective role during cerebral ischemia in mice. Although both studies strongly support a protective role of Cav-1 in mouse models of tissue ischemia, the clinical significance of these findings still remains to be determined. Taken together, the results of these studies suggest that endothelial Cav-1 is essential for optimal recovery after ischemia-induced injury in mouse models of ischemia. However, clinical significance of these observation s remains to be elucidated.

## Does endothelial Cav-2 play a role in disease?

Original studies revealed a hyperproliferative phenotype in the lung of Cav-2 KO mice and suggested ECs as a major hyperproliferating cell type [22]. Interestingly, the identical phenotype was in the lungs of Cav-1 KO mice [48]. However, the expression level of Cav-2 in the lung of Cav-1 KO mice diminishes to ca. 5% of the respective expression level in WT mice. In addition, the remaining Cav-2 does not target to plasma membrane lipid raft/caveolar domains and thus loses any function which is dependent on plasma membrane caveolae targeting. In contrast, the expression level of Cav-1 in the lung of Cav-2 KO mice is only reduced to 50%, and Cav-1 heterozygotes expressing comparable level of Cav-1 to that observed in Cav-2 KO mice do not develop the hyperproliferative phenotype. Overall, these findings implicate the lack of Cav-2 rather than Cav-1 as the direct cause for the hyperproliferative phenotype in the lung. Interestingly, the major hyperproliferating cell type in the lung appears to be VEGF-R2 (known as Flk-1 in mouse) positive and because Flk-1 is predominantly expressed in ECs, this observation suggests that Cav-2 may negatively regulate lung microvascular EC proliferation. The mechanistic nature of this seemingly EC-specific hyperproliferation in the lung requires further investigation. Interestingly, as previously discussed for Cav-1, the same EC-specific hyperproliferative phenotype observed in Cav-1 KO mice has been implicated in specific diseases such as pulmonary hypertension. Moreover, in addition to Cav-1, Cav-2 was also reduced in rats with moncrotaline-induced pulmonary hypertension [79]. Remarkably, as determined in a rat model of chemically-induced pulmonary hypertension, similar increases in tyrosine phosphorylated STAT3, and the expression levels of cyclin D1 and D3 were observed in whole lung homogenates from Cav-1 and Cav- 2 KO mice [79]. However, unlike in Cav-1 KO mice, the possibility that Cav-2 KO mice have pulmonary hypertension was not examined. Nevertheless, based on previously discussed dependence of Cav-2 protein stability and plasma membrane lipid raft/caveolar targeting on Cav-1, it is possible that reduction/loss of Cav-2 rather than Cav-1 could be more directly responsible for hyperactivation of the STAT3 pathway, increased expression of cyclins D1 and D3, and pulmonary hypertension in mice and possibly rats with chemically-induced pulmonary hypertension. Further studies testing the possibility of pulmonary hypertension in Cav-2 KO mice will be necessary. In addition to these in vivo studies, several recently reported studies using cultured ECs suggest the importance of Cav-2 in regulating EC proliferation and TGF-β mediated signaling and function in ECs. Specifically, to test if the hyperproliferative phenotype involving Flk-1 positive cells may indicate that Cav-2 plays a negative role in EC proliferation, we have compared proliferation potential in lung ECs isolated from WT and Cav-2 KO mice. The results of these studies revealed that Cav-2 suppresses lung microvascular EC proliferation by inhibiting extracellular signal regulated kinase 1/2 (ERK1/2) phosphorylation, increased expression of cyclin-dependent kinase (cdk) inhibitors p16INK4 and p27Kip1 and activation (hypophosphorylation) of the retinoblastoma (Rb) protein, resulting in a reduced cell cycle progression [137]. Consistent with our data in lung ECs, more recently, another group has also reported anti-proliferative function of Cav-2 in a rat prostate EC cell line (YPEN-1) [138]. These data suggests that in addition to the lung, Cav-2 can also inhibit EC proliferation from other organs such as the prostate. Our most recent findings suggest that the role of Cav-2 in regulating lung micro-vascular EC proliferation is more complex and context-specific [139]. Specifically, we have shown that Cav-2 may be a physiological inhibitor of anti-proliferative function and signaling of TGF-β in ECs. Mechanistically, Cav-2 inhibits anti-proliferative action of TGF-β by suppressing Alk5/Smad2/3 pathway manifested by reduced magnitude and length of TGF-β-induced Smad2/3 phosphorylation as well as activation of Alk5/Smad2/3 target genes, plasminogen activator inhibitor-1 and collagen type I in Cav-2-positive ECs. Because EC responses to TGF-β could be important for various processes such as angiogenesis, atherosclerosis, or even for any disorders involving fibrosis due to endothelial to mesenchymal transition, our data with exaggerated response of Cav-2 KO ECs to TGF-β may imply that Cav- 2 plays a role in some of the above mentioned disorders. However, studies addressing the role of endothelial Cav-2 in regulating TGF-β signaling and function in vivo will be necessary.

## Conclusions, Future Directions, and Clinical Significance

The literature reviewed here suggests that endothelial caveolae and their major coat protein Cav-1 play an important role in regulating cardiovascular and pulmonary function and associated disorders. Remarkably, specific loss of Cav-1 in ECs appears to be responsible for many of pathologies reported in Cav-1 KO including pulmonary hypertension and cardiac hypertrophy (Figure 1). Moreover, endothelial Cav-1 may promote pathological processes such as acute lung injury, atherosclerosis, or pathological angiogenesis associated with cancer and inflammation. Also, clinical evidence supports the importance of endothelial Cav-1 deficiency in development of pulmonary hypertension in patients. Remarkably, delivery of a cell-permeable peptide containing CSD proved to effectively prevent the development of pulmonary hypertension, right ventricular hypertrophy, and pulmonary artery medial hypertrophy in a monocrotaline-induced pulmonary hypertension rat model [83]. Thus, development of therapeutic approaches involving endothelium-specific delivery of Cav-1 mimicking peptides or gene therapy restoring Cav-1 expression in endothelium of patients with pulmonary hypertension could be of clinical significance. In addition to pulmonary hypertension, administration of a cell-permeable peptide containing CSD also suppressed tumor progression in mice by blocking microvascular permeability [140]. Thus specific delivery of Cav-1 mimetic peptides to tumor blood vessels of cancer patients could potentially be exploited in antitumor therapy.

In addition to Cav-1, endothelial Cav-2 may play a role in pulmonary and possibly other diseases. However, additional experimental and clinical evidence will be required to determine specific importance of endothelial Cav-2 in disease. Despite considerable progress, many unresolved issues still remain with respect to caveolae and caveolins, although the role of endothelial Cav-1 in pulmonary and vascular diseases seems unquestioned. In particular, development of tools allowing endothelial-specific targeting or mimicking Cav-1 and possibly Cav-2, will be important because of the ubiquitous expression of Cav-1 and -2 proteins.
